# Deep-Sea Fungi Could Be the New Arsenal for Bioactive Molecules

**DOI:** 10.3390/md18010009

**Published:** 2019-12-20

**Authors:** Muhammad Zain ul Arifeen, Yu-Nan Ma, Ya-Rong Xue, Chang-Hong Liu

**Affiliations:** State Key Laboratory of Pharmaceutical Biotechnology, School of Life Sciences, Nanjing University, Nanjing 210023, China; m.z.arifeen@gmail.com (M.Z.u.A.); mayunan994727@163.com (Y.-N.M.); xueyr@nju.edu.cn (Y.-R.X.)

**Keywords:** deep-sea, extreme, ecosystem, fungi, bioactive compounds, secondary metabolites

## Abstract

Growing microbial resistance to existing drugs and the search for new natural products of pharmaceutical importance have forced researchers to investigate unexplored environments, such as extreme ecosystems. The deep-sea (>1000 m below water surface) has a variety of extreme environments, such as deep-sea sediments, hydrothermal vents, and deep-sea cold region, which are considered to be new arsenals of natural products. Organisms living in the extreme environments of the deep-sea encounter harsh conditions, such as high salinity, extreme pH, absence of sun light, low temperature and oxygen, high hydrostatic pressure, and low availability of growth nutrients. The production of secondary metabolites is one of the strategies these organisms use to survive in such harsh conditions. Fungi growing in such extreme environments produce unique secondary metabolites for defense and communication, some of which also have clinical significance. Despite being the producer of many important bioactive molecules, deep-sea fungi have not been explored thoroughly. Here, we made a brief review of the structure, biological activity, and distribution of secondary metabolites produced by deep-sea fungi in the last five years.

## 1. Deep-Sea Fungi: A Novel Source of Bioactive Molecules

Antibiotics and antifungal drugs are the most commonly used drugs in the world, but their role in treating human diseases has been greatly reduced due to the development of pathogen resistance against these drugs. Scientists are now looking for new, untapped and renewable resources for the isolation of novel compounds to with clinical importance. Despite the fact that the ocean provides habitats to a huge number of microbes, both fungi and bacteria for thousands of years, the microbes of these extreme ecosystems and their potential for new drug discovery have not yet been fully realized due to methodological and technical limitations. Fungi are the most diverse and abundant eukaryotic organisms on the planet, and their presence in all possible extreme ecosystems make them an ideal source for investigations of new drug development. Scientists are interested in the extraction of novel and unique natural products, having clinical importance, from different organisms living in the extreme environments. In addition to terrestrial extreme environments, the ocean could also be considered a good reservoir of bioactive metabolites [1,2,3,4]. Fungi living in the deep-sea environments are known to produce novel bioactive compounds. Although, it is not fully understood why the fungi living in the extreme environments produce unique and novel products, it is assumed that fungal genome has evolved to make necessary adjustments in order to sustain life in such harsh conditions and might be involved in chemical defense and communication [5].

The ocean is considered to be one of the most diverse ecosystems. Compared to terrestrial and coastal ecosystems, the deep-sea (water depths below 1000 m) has a variety of extreme environments, such as temperatures ranging from 0 to 400 °C, lack of light and oxygen, high hydrostatic pressure up to 400 atm, and limited supply of nutrient substrates, making these habitats extremely difficult for life [6,7]. In order to inhabit such extreme ecosystems, organisms should have the potential to adjust to these conditions with different mechanism, such as regulating temperature, pH, and solute concentration, as well as the production of biomolecules to control DNA, protein, and lipid damage. This may be why microorganisms growing in these environments produce special metabolites.

Previously, drug investigators mainly considered bacteria, especially actinomycetes, as an important source of antifungal and antibacterial drugs. Cephalosporin C was the first compound derived from the marine fungus *Cephalosporium* sp. in 1949. After that, a number of important drugs— for instance, polyketide griseofulvin, terpenoid fusidic acid, cephalosporins, etc.—have been isolated from the marine fungi. Despite being the source of such important products, deep-sea fungi have not received full attention [8]. With the increasing demand for new drugs, scientists are now looking for new and unexplored resources for bioactive compounds, and the deep-sea consists of some extreme ecosystems that are worth exploring for new metabolites. Studies about isolating new bioactive molecules from marine environments are growing at an increasing rate, and hundreds of new compounds are reported every year; for instance, in 2017, a total of 448 new compounds were reported [9].

In this review, we present an overview of all those new and important bioactive metabolites isolated from deep-sea fungi during the last five years. We include only those molecules which were extracted from the deep-sea fungi associated with some kind of extreme environments, irrespective of its isolation from terrestrial counterparts, while all those compounds were excluded which were isolated from marine fungi and were not associated with extreme environments. This review will benefit all those who are interested in extreme-marine-environment fungi and their bioactive molecules. For more detailed information about other important secondary metabolites extracted from marine fungi, one should refer to our previous review papers [10,11,12].

## 2. Bioactive Compounds from Deep-Sea Fungi

According to the literature survey, we found 151 novel bioactive compounds isolated from marine fungi extracted from different extreme environments in the last five years. The majority of these compounds were isolated from two fungal genera i.e., *Penicillium* (63, 41.2% of the total compounds) and *Aspergillus* (43, 28.1% of the total compounds). Table 1 lists the detail of these compounds, which fall into different categories according to their structure.

### 2.1. Polyketide Compounds

**Figure 1:**
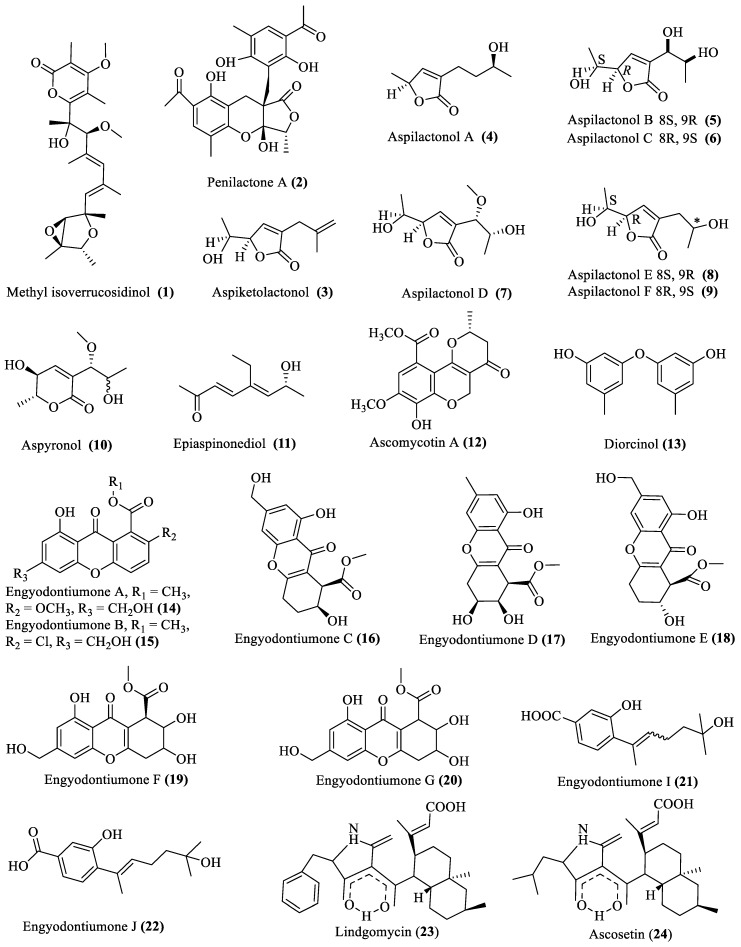
Structures of polyketide secondary metabolites obtained from deep-sea fungi.

Twenty-four polyketide compounds (**1**–**24**; Figure 1) with important biological activities were isolated from fungi extracted from different deep-sea environments. Among them, compounds **1** and **2** were isolated from *Penicillium* spp., which showed antibiotic activity (MIC of 32 μg/mL against *Bacillus subtilis*) and nuclear factor NF-kB inhibition activity, respectively [13,14]. Compounds **3**–**11** were from *Aspergillus* sp. 16-02-1, which exhibited cytotoxicity (with a 10%–80% inhibition rate at 100 μg/mL against various cancer cell lines i.e., K562, HL-60, HeLa, and BGC-823) [15]. Similarly, compounds **12**–**24** were isolated from the species belonging to *Ascomycetes*, *Engyodontium*, and *Lindgomycetaceae*, out of which compounds **12**–**13** and **23**–**24** showed strong antibiotic activities against *Bacillus subtilis*, *Acinetobacter baumannii*, *Escherichia coli*, *Staphylococcus aureus*, *Enterococcus faecalis*, *Staphylococcus epidermidis*, and *Propionibacterium acnes*, while compounds **14**–**22** exhibited strong cytotoxic activity (IC_50_ 4.9 µM) against U937 cells (Table 1) [16,17,18].

### 2.2. Nitrogen-Containing Compounds

**Figure 2:**
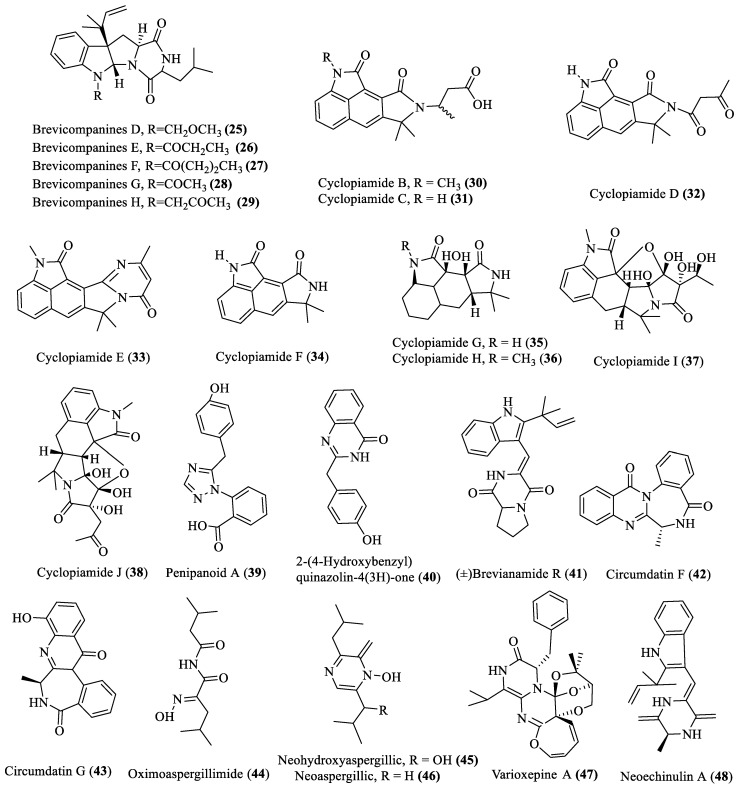
Bioactive alkaloid compounds isolated from deep-sea fungi.

Twenty-four novel alkaloid-bioactive compounds (**25**–**48**; Figure 2) have been reported from deep-sea fungi since 2013, out of which compounds **25**–**40** were isolated from *Penicillium* spp., and showed cytotoxic activities against BV2 cell (IC_50_ of 27–45 µg/mL), brine shrimp (IC_50_ of 14.1 to 38.5 µg/mL), SMMC-7721 (IC_50_ of 54.2 µM), BEL-7402 ((IC_50_ of 17.5 µM), and BEL-7402 (IC_50_ of 19.8 µM) [19,20,21]. Compounds **41**–**46** were identified from *Aspergillus* spp., in which compounds **41** and **45**–**46** displayed antibiotic activity (MIC of 30 to 40 µg/mL) against BCG, *Candida albicans*, *Bacillus subtilis*, *Staphylococcus aureus*, *Pseudomonas aeruginosa*, *Bacillus cereus*, *Klebsiella pneumoniae*, and *Escherichia coli*, while compounds **47** and **48** were extracted from other genera and showed antimicrobial activity (MIC between 16 and 64 µg/mL against *Escherichia coli*, *Aeromonas hydrophila*, *Micrococcus luteus*, *Staphylococcus aureus*, *Vibrio anguillarum*, *Vibrio harveyi*, and *Vibrio parahaemolyticus*) and cytotoxic activity against human cervical carcinoma HeLa, respectively [22,23,24,25,26].

### 2.3. Polypeptides

**Figure 3:**
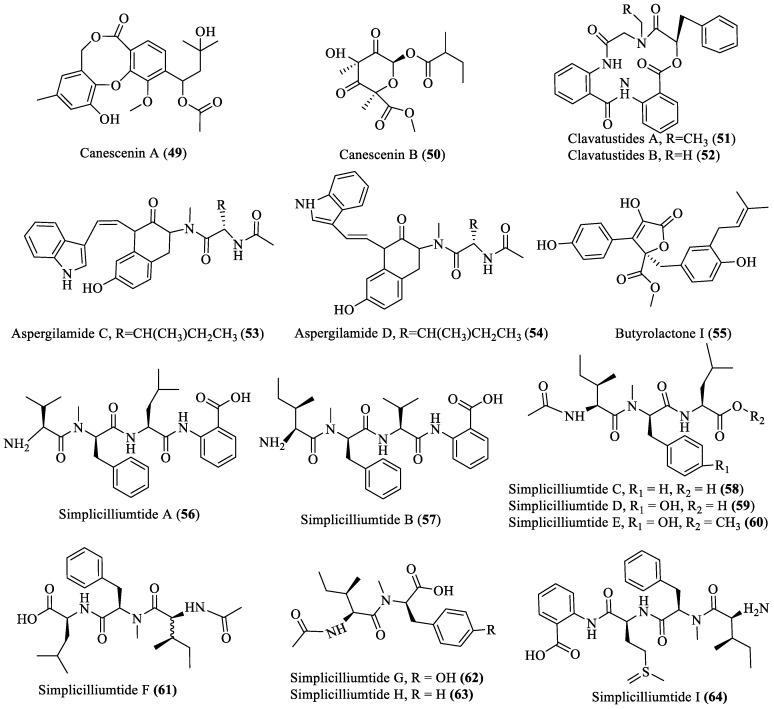

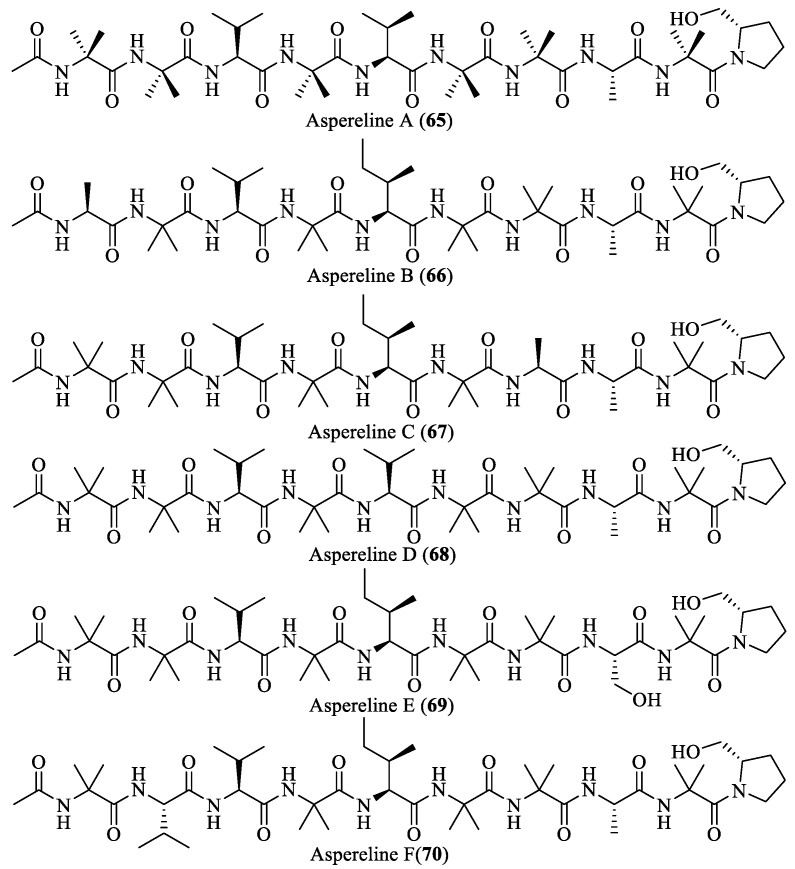
Bioactive polypeptides isolated from deep-sea fungi.

Twenty-two polypeptides with novel structures (**49**–**70**; Figure 3) were reported from fungi inhabiting different marine environments during 2013–2019. Compounds **49** and **50** were isolated from *Penicillium canescens* and displayed antibiotic activity against *Bacillus amyloliquefaciens* and *Pseudomonas aeruginosa* at 100 µM, while compounds **51**–**55** were extracted from *Aspergillus* spp., in which **51**–**54** showed cytotoxic activity (IC_50_ of 15–25 μg/mL) against HepG2, SMMC-7721, Bel-7402, and human glioma U87 cell lines, while compound **55** showed inhibitory effects (IC_50_ value of 5.11 μmol/L) against *Mycobacterium tuberculosis* protein tyrosine phosphatase B (MptpB) [27,28,29,30]. However, compounds **56**–**64**, which were obtained from *Simplicillium obclavatum,* and **65**–**70**, obtained from *Trichoderma asperellum,* displayed cytotoxicity (IC_50_ of 39.4–100 µM) against human leukemia HL-60 and K562 cell lines and antibiotic activity (IC_50_ of 39.4–100 µM) against Gram-positive bacteria (e.g., *Bacillus amyloliquefaciens*, *Staphylococcus aureus*) and Gram-negative bacteria (e.g., *Pseudomonas aeruginosa* and *Escherichia coli*), respectively [28,31].

### 2.4. Ester and Phenolic Derivatives

**Figure 4:**
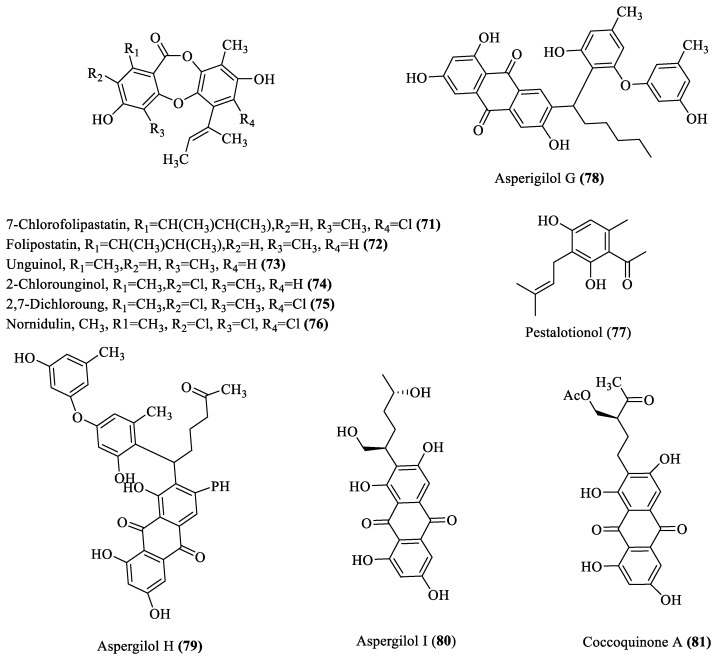
Ester and phenolic derivatives obtained from deep-sea fungi.

Six new ester derivatives (**71**–**76**; Figure 4) were extracted from *Aspergillus ungui* NKH-007 and showed inhibition of sterol O-acyltransferase (SOAT) enzymes in Chinese hamster ovary (CHO) cells and are thus considered to be good candidates for an anti-atherosclerotic agent [32]. Five new phenolic compounds (**77**–**81**; Figure 4) isolated from *Penicillium* sp. and *Aspergillus versicolor* showed potent activity against *Staphylococcus aureus* and *Bacillus subtilis*, with MIC values of 2–8 μg/mL [33,34]. However, compounds **78**–**81** expressed antiviral activity toward HSV-1, with EC_50_ values of 3.12–6.25 μM [34].

### 2.5. Piperazine Derivatives

**Figure 5:**
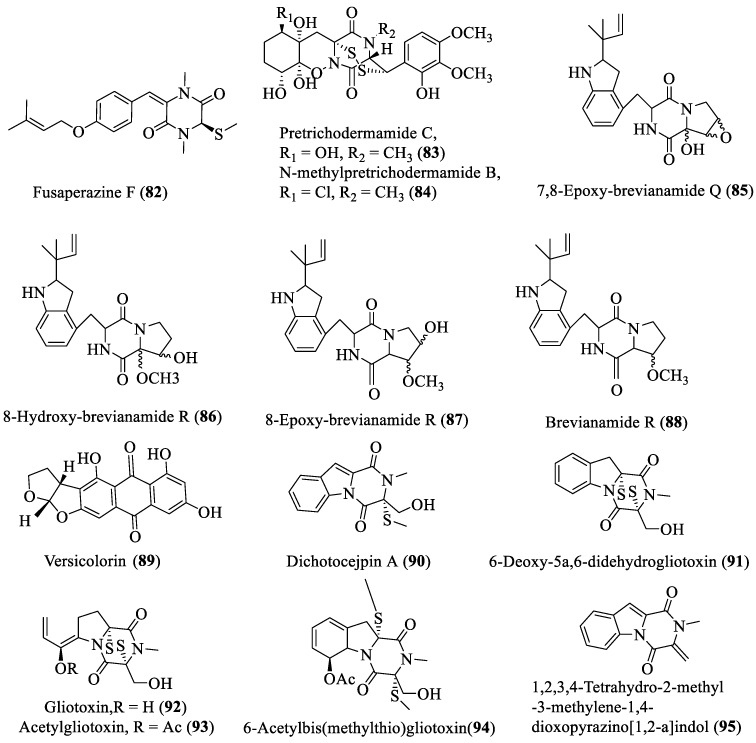
Piperazine derivatives isolated from deep-sea fungi.

Fourteen new piperazine derivatives (**82**–**95**; Figure 5) reported from marine fungi during the last five years. These derivatives were isolated from genera of *Penicillium*, *Aspergillus*, and *Dichotomomyces* collected from deep-sea sediments. Compounds **82**–**84** showed strong cytotoxicity with IC_50_ of 1.7 and 2 µM against K562 and mouse lymphoma cell line, respectively; similarly, compounds **91**–**95** also showed strong cytotoxic activity [35,36,37]. Compounds **85**–**89** showed antibacterial activity against *Staphylococcus aureus* with the MIC values of 6.25–12.5 µg/mL [21]. The new compound **90** also showed stronger inhibition activity against α-glucosidase with IC_50_ value of 138 µM [37].

### 2.6. Terpenoid Compounds

**Figure 6:**
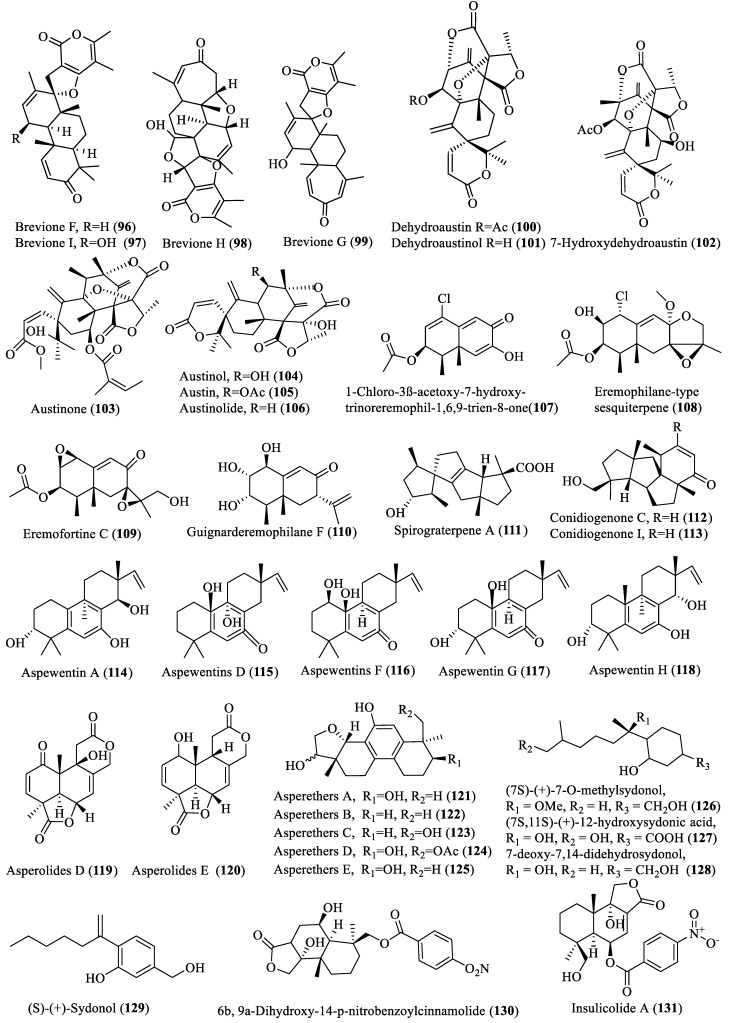
Structures of terpenoid secondary metabolites obtained from deep-sea fungi.

Thirty-six new and important bioactive terpenoids (**96**–**131**; Figure 6) have been isolated from marine fungi extracted from the deep-sea sediments since 2013. Compounds **96**–**113** were isolated from *Penicillium* spp., while compounds **114**–**131** were extracted from *Aspergillus* spp. Breviones (**96**–**99**), isolated from the deepest sediment-derived fungus *Penicillium* sp. (5115 m depth), displayed diverse activities, such as cytotoxicity against HeLa, MCF-7, and A549 cells with IC_50_ values of 7.44 to 32.5 µM, respectively, and growth inhibition of HIV-1 with EC_50_ value of 14.7 µM against C8166 cells [22,38]. Compounds **100**–**110** showed antibiotic and inhibition activities against silkworm, while 20-nor-isopimarane diterpenoids, including aspewentins (**114**–**118**), asperethers (**121**–**125**), asperoloids (**119**–**120**), and compounds **130** and **131**, showed cytotoxic activities [33,39,40,41,42,43,44,45]. However, the spirocyclic diterpenes (**111**–**113)** exhibited strong anti-allergic effect with 18% inhibition at 20 μg/mL [46]. Interestingly, four new compounds (**126**–**129**) were extracted from hydrothermal vent-derived *Aspergillus sydowii*, through activation of a new pathway for secondary metabolite production by the addition of a 5-azacytidine (a DNA methyltransferase inhibitor). These compounds showed anti-inflammatory and antidiabetic activities and are thus the first secondary metabolites isolated from fungi which have both antidiabetic and anti-inflammatory activities [47].

### 2.7. Other Unrelated Compounds

**Figure 7:**
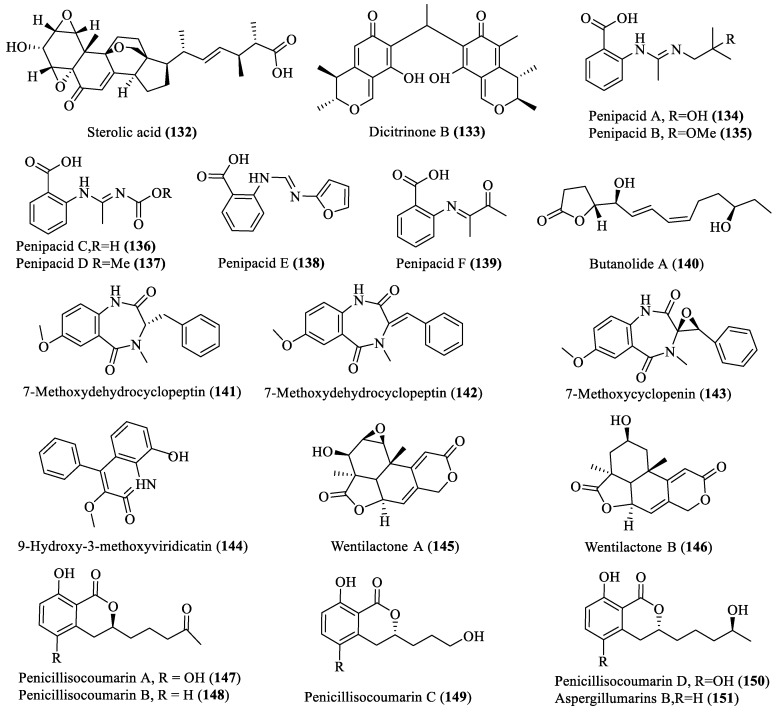
Bioactive metabolites derived from deep-sea fungi.

Twenty secondary metabolites with different structures were isolated from deep-sea fungi, mainly from *Penicillium* spp. and *Aspergillus* spp. (**132**–**151**; Figure 7). Penipacids A–F (**134**–**139**), polyoxygenated sterol (**132**), dicitrinone B (**133**) and butanolide A (**140**), which were isolated from deep-sea sediments-derived *Penicillium* spp., showed cytotoxic activities against RKO, MCF-7, PTP1B and A375 cancer cell lines with IC_50_ values of 8.4–28.4 µM [38,42,48,49]. Similarly, four isocoumarins, penicillisocoumarin A–D (**147**–**150**), and an isocoumarins aspergillumarin B (**151**) were also isolated from *Penicillium* which showed weak antibacterial activities [33]. Four antibiotic cyclopenin derivatives compounds (**141**–**144**) and a series of antitumor wentilactones (**145**,**146**) were isolated from *Aspergillus* spp. [50,51].

## 3. Conclusions and Perspective

The results of current studies indicate that the deep-sea extreme environmental fungi are one of the rich and unexploited sources of important medicinal lead compounds. Most of the fungi (e.g., *Penicillium* spp. and *Aspergillus* spp.) living in the extreme environments of the deep-sea have the potential to synthesize new bioactive compounds. However, the research on deep-sea fungi and their metabolites is very limited due to the difficulty of sampling and the limitation of culture technology. Thanks to the advances in genome technology and the implementation of the deep-sea drilling program, novel compounds with great biological activities are expected from these fungi in the near future. From the literature review, we can say these fungi from the extreme environments have the potential to produce clinically important natural products. The compounds we discussed in this review show strong bioactivities and might have the potential to be a future anticancer drug. Among them, terpenoid derivatives were the most important and abundant compound category which were mainly isolated from deep-sea derived *Penicillium* spp. and *Aspergillus* spp. This class of compounds showed strongest antibiotic and cytotoxic activities as compared to other classes of compounds and has the potential to be a future candidate for anticancer drugs, especially brevione, which was isolated from the deepest part of the sea and showed the strongest cytotoxic activity.

## Figures and Tables

**Table 1:** Secondary metabolites extracted from deep-sea fungi during 2013–2019.

Metabolites	Fungal Species	Source	Location	Depth (m) ^*^	Bioactivity	Ref.
**Polyketide**						
Methyl-isoverrucosidinol **(1)**	*Penicillium* sp. Y-50-10	Sulfur-rich Sediment	hydrothermal vent, Taiwan	_	Antibiotic	[13]
Penilactone A **(2)**	*Penicillium crustosum* PRB-2	Sediment	Prydz Bay, Antarctica	526	NF-kB inhibition	[14]
Aspiketolactonol **(3)** Aspilactonols A–F **(4-9)** Aspyronol **(10)** Epiaspinonediol **(11)**	*Aspergillus* sp. 16-02-1	Hydrothermal vent water	Lau Basin, Southwest Pacific Ocean,	2255	Cytotoxic	[15]
Ascomycotin A **(12)** Diorcinol **(13)**	*Ascomycota* sp. Ind19F07	Sediment	Indian Ocean	3614	Antibiotic	[16]
Engyodontiumones A–J **(14-22)**	*Engyodontium album* DFFSCS021	Sediment	South China Sea	3739	Cytotoxic	[18]
Lindgomycin **(23)** Ascosetin **(24)**	*Lindgomycetaceae* strains KF970 and LF327	Sediment	Greenland Sea, Baltic Sea	3650	Antibiotic	[17]
**Nitrogen-containing compounds**						
Brevicompanines D–H **(25-29)**	*Penicillium* sp. F1	Sediment	_	5080	LPS-induced inflammation	[22]
Cyclopiamide B–J **(30-38)**	*Penicillium commune* DFFSCS026	Sediment	South China Sea	3563	Cytotoxic	[24]
Penipanoid A **(39)** Quinazolinone **(40)**	*Penicillium paneum* SD-44	Sediment	South China Sea	201	Cytotoxic	[23]
(±) Brevianamide R **(41)**	*Aspergillus* versicolor MF180151	Sediment	Bohai Sea, China	_	Antibacterial	[21]
Circumdatin F and G **(42-43)**	*Aspergillus westerdijkiae* SCSIO 05233	Sediment	South China Sea	4593	Cytotoxic	[20]
Oximoaspergillimide **(44)** Neohydroxyaspergillic **(45)** Neoaspergillic **(46)**	*Aspergillus* sp. (CF07002)	Water	Pacific Ocean off the coast of Panama		Cytotoxic Antibiotic	[19]
Varioxepine A **(47)**	*Paecilomyces variotii* EN-291	Deep sea water	_	_	Antibiotic	[26]
Neoechinulin A **(48)**	*Microsporum* sp. (MFS-YL)	Red alga	Guryongpo, Korea	_	Cytotoxic	[25]
**Polypeptide**						
Canescenin A and B **(49-50)**	*Penicillium canescens* SCSIO z053	Water	East China Sea	2013	Antibacterial	[27]
Clavatustide A and B **(51-52)**	*Aspergillus clavatus* C2WU	Hydrothermal vent crab	Taiwan Kueishantao	_	Cytotoxic	[29]
Aspergillamides C and D **(53-54)** Butyrolactone I **(55)**	*Aspergillus terreus* SCSIO 41008	Sponge	Guangdong, China	_	Cytotoxic Antibiotic	[30]
Simplicilliumtides A–I **(56-64)**	*Simplicillium obclavatum* EIODSF 020	Sediment	East Indian Ocean	4571	Cytotoxic	[31]
Asperelines A–F **(65-70)**	*Trichoderma asperellum*	Sediment	Antarctic Penguin Island	_	Antibiotic	[28]
**Esters**						
7-chlorofolipastatin **(71)** Folipostatin B **(72)** Unguinol **(73)** 2-chlorounginol **(74)** 2,7-dichlorounguinol **(75)** Nornidulin **(76)**	*Aspergillus ungui* NKH-007	Sediment	Suruga Bay, Japan	_	Anti-atherosclerotic Cytotoxic Antibiotic	[32]
**Phenolic**						
Pestalotionol **(77)**	*Penicillium* sp. Y-5-2	Hydrothermal vent water	Kueishantao off Taiwan	_	Antibiotic	[33]
Aspergilol G–I **(78-80)** Coccoquinone A **(81)**	*Aspergillus versicolor* SCSIO 41502	Sediment	South China Sea	2326	Anti-HSV-1 Antioxidant Antifouling	[34]
**Piperazine**						
Fusaperazine F **(82)**	*Penicillium crustosum* HDN153086	Sediment	Prydz Bay, Antarctica	_	Cytotoxic	[35]
N-methyl-pretrichodermamide B **(83)** Pretrichodermamide C **(84)**	*Penicillium* sp. (WN-11-1-3-1-2)	Hypersaline sediment	Wadi El-Natrun, Egypt	_	Cytotoxic	[36]
(±) 7,8-epoxy-brevianamide Q **(85)** (±) 8-hydroxy-brevianamide R **(86)** (±) 8-epihydroxy-brevianamide R **(87)** Brevianamide R **(88)** Versicolorin B **(89)**	*Aspergillus versicolor* MF180151	Sediment	Bohai Sea, China	_	Antibiotic	[21]
Dichotocejpins A **(90)** 6-deoxy-5a,6-didehydrogliotoxin **(91)** Gliotoxin **(92)** Acetylgliotoxin **(93)** 6-acetylbis(methylthio)-gliotoxin **(94)** 1,2,3,4-tetrahydro-2-methyl-3-methylene-1,4-dioxopyrazino [1,2-a] indole **(95)**	*Dichotomomyces cejpii* FS110	Sediment	South China Sea	3941	α-Glucosidase inhibition Cytotoxic	[37]
**Terpenoid**						
Brevione F–I **(96-99)**	*Penicillium* sp. (MCCC 3A00005)	Sediment	Pacific Ocean	5115	Cytotoxic HIV-1 inhibition	[22,38]
Dehydroaustin **(100)** Dehydroaustinol **(101)** 7-hydroxydehydroaustin **(102)** Austinone **(103)** Austinol **(104)** Austin **(105)** Austinolide **(106)**	*Penicillium* sp. Y-5-2	Hydrothermal vent water	Kueishantao off Taiwan	8	Antibacterial Anti-insectal	[33]
1-chloro-3β-acetoxy-7-hydroxytrinoreremophil-1,6,9-trien-8-one **(107)** Eremophilane-type sesquiterpenes **(108)** Eremofortine C **(109)**	*Penicillium* sp. PR19N-1	Sediment	Prydz Bay, Antarctica	526	Cytotoxic	[40,41]
Guignarderemophilane F **(110)**	*Penicillium* sp. S-1-18	Sediment	Antarctic	1393	Antibacterial	[42]
Spirograterpene A **(111)** Conidiogenone C and I **(112-113)**	*Penicillium granulatum* MCCC 3A00475	Water	Prydz Bay of Antarctica	2284	Antiallergic	[46]
Aspewentin A and D–H **(114-118)** Asperethers A–E **(121-125)** Asperolides D and E **(119-120)**	*Aspergillus wentii* SD-310	Sediment	South China Sea	2038	Antimicrobial Cytotoxic Anti-inflammatory	[39,43,44]
(7S)-(+)7-O-methylsydonol **(126)** (7S,11S)-(+)-12-hydroxysydonic acid **(127)** 7-deoxy-7,14-didehydrosydonol **(128)** (S)-(+)-sydonol **(129)**	*Aspergillus sydowii*	Sediment	Hsinchu, Taiwan	_	Anti-inflammatory	[47]
6b,9a-dihydroxy-14-p-nitrobenzoylcinnamolide **(130)** Insulicolide A **(131)**	*Aspergillus ochraceus* Jcma1F17	Marine alga *Coelarthrum* sp.	South China Sea	_	Antiviral Cytotoxic	[45]
**Other compounds**						
Sterolic acid **(132)**	*Penicillium* sp. MCCC 3A00005	Sediment	East Pacific Ocean	5115	Cytotoxic	[38]
Dicitrinone B **(133)**	*Penicillium citrinum*	Sediment	Langqi Island, Fujian, China	_	Antitumor	[49]
Penipacids A–F **(134-139)**	*Penicillium paneum* SD-44	Sediment	South China Sea	_	Cytotoxic	[48]
Butanolide A **(140)**	*Penicillium* sp. S-1-18	Sediment	Antarctic seabed	1393	Cytotoxic	[42]
7-Methoxycyclopeptin **(141)** 7-Methoxy dehydro cyclopeptin **(142)** 7-Methoxy cyclopenin **(143)** 9-Hydroxy-3-methoxyviridicatin **(144)**	*Aspergillus versicolor* XZ-4	Hydrothermal vent crab	Kueishantao, Taiwan		Antibiotic	[50]
Wentilactone A and B **(145-146)**	*Aspergillus dimorphicus* SD317	Sediment	South China Sea	2038	Antitumor	[51]
Penicillisocoumarin A–D **(147-150)** Aspergillumarins B **(151)**	*Penicillium* sp. Y-5-2	Hydrothermal vent water	Kueishantao off Taiwan	8	Antibacterial	[33]

* Depth represents water depth below the surface.

## References

[1] König G.M., Kehraus S., Seibert S.F., Abdel-Lateff A., Müller D. (2006). Natural products from marine organisms and their associated microbes. Chem. Bio. Chem..

[2] Chen G., Wang H.-F., Pei Y.-H. (2014). Secondary metabolites from marine-derived microorganisms. J. Asian Nat. Prod. Res..

[3] Agrawal S., Adholeya A., Deshmukh S.K. (2016). The pharmacological potential of non-ribosomal peptides from marine sponge and tunicates. Front. Pharmacol..

[4] Deshmukh S.K., Prakash V., Ranjan N. (2017). Recent advances in the discovery of bioactive metabolites from *Pestalotiopsis*. Phytochem. Rev..

[5] Deshmukh S.K., Prakash V., Ranjan N. (2018). Marine fungi: A source of potential anticancer compounds. Front. Microbiol..

[6] Danovaro R., Corinaldesi C., Dell’Anno A., Snelgrove P.V. (2017). The deep-sea under global change. Curr. Biol..

[7] Barone G., Varrella S., Tangherlini M., Rastelli E., Dell’Anno A., Danovaro R., Corinaldesi C. (2019). Marine fungi: Biotechnological perspectives from deep-hypersaline anoxic basins. Diversity.

[8] Hamilton-Miller J. (2008). Development of the semi-synthetic *penicillins* and *cephalosporins*. Int. J. Antimicrob..

[9] Carroll A.R., Copp B.R., Davis R.A., Keyzers R.A., Prinsep M.R. (2019). Marine natural products. Nat. Prod. Rep..

[10] Wang Y.-T., Xue Y.-R., Liu C.-H. (2015). A brief review of bioactive metabolites derived from deep-sea fungi. Mar. Drugs.

[11] Arifeen M.Z.U., Liu C.-H. (2018). Novel enzymes isolated from marine-derived fungi and its potential applications. United J. Biochem. Biotechnol..

[12] Arifeen M.Z.U., Xue Y.-R., Liu C.-H. (2019). Deep-sea fungi: Diversity, enzymes, and bioactive metabolites. Fungi in Extreme Environments: Ecological Role and Biotechnological Significance.

[13] Pan C., Shi Y., Auckloo B., Chen X., Chen C.-T., Tao X., Wu B. (2016). An unusual conformational isomer of verrucosidin backbone from a hydrothermal vent fungus, *Penicillium* sp. Y-50-10. Mar. Drugs.

[14] Wu G., Ma H., Zhu T., Li J., Gu Q., Li D. (2012). Penilactones A and B, two novel polyketides from Antarctic deep-sea derived fungus *Penicillium crustosum* PRB-2. Tetrahedron.

[15] Chen X.-W., Li C.-W., Cui C.-B., Hua W., Zhu T.-J., Gu Q.-Q. (2014). Nine new and five known polyketides derived from a deep sea-sourced *Aspergillus* sp. 16-02-1. Mar. Drugs.

[16] Tian Y.-Q., Lin X.-P., Liu J., Kaliyaperumal K., Ai W., Ju Z.-R., Yang B., Wang J., Yang X.-W., Liu Y. (2015). Ascomycotin A, a new citromycetin analogue produced by *Ascomycota* sp. Ind19F07 isolated from deep sea sediment. Nat. Prod. Res..

[17] Wu B., Wiese J., Labes A., Kramer A., Schmaljohann R., Imhoff J. (2015). Lindgomycin, an unusual antibiotic polyketide from a marine fungus of the *Lindgomycetaceae*. Mar. Drugs.

[18] Yao Q., Wang J., Zhang X., Nong X., Xu X., Qi S. (2014). Cytotoxic polyketides from the deep-sea-derived fungus *Engyodontium album* DFFSCS021. Mar. Drugs.

[19] Cardoso-Martínez F., de la Rosa J.M., Díaz-Marrero A.R., Darias J., D’Croz L., Cerella C., Diederich M., Cueto M. (2015). Oximoaspergillimide, a fungal derivative from a marine isolate of *Aspergillus* sp.. Eur. J. Org. Chem..

[20] Fredimoses M., Zhou X., Ai W., Tian X., Yang B., Lin X., Xian J.-Y., Liu Y. (2015). Westerdijkin A, a new hydroxyphenylacetic acid derivative from deep sea fungus *Aspergillus westerdijkiae* SCSIO 05233. Nat. Prod. Res..

[21] Hu J., Li Z., Gao J., He H., Dai H., Xia X., Liu C., Zhang L., Song F. (2019). New diketopiperazines from a marine-derived fungus strain *Aspergillus versicolor* MF180151. Mar. Drugs.

[22] Zhang X., Li S.-J., Li J.-J., Liang Z.-Z., Zhao C.-Q. (2018). Novel natural products from extremophilic fungi. Mar. Drugs.

[23] Li C.-S., An C.-Y., Li X.-M., Gao S.-S., Cui C.-M., Sun H.-F., Wang B.-G. (2011). Triazole and dihydroimidazole alkaloids from the marine sediment-derived fungus *Penicillium paneum* SD-44. J. Nat. Prod..

[24] Xu X., Zhang X., Nong X., Wei X., Qi S. (2015). Oxindole alkaloids from the fungus *Penicillium commune* DFFSCS026 isolated from deep-sea-derived sediments. Tetrahedron.

[25] Wijesekara I., Li Y.-X., Vo T.-S., Van Ta Q., Ngo D.-H., Kim S.-K. (2013). Induction of apoptosis in human cervical carcinoma HeLa cells by neoechinulin A from marine-derived fungus *Microsporum* sp.. Process Biochem..

[26] Zhang P., Mandi A., Li X.-M., Du F.-Y., Wang J.-N., Li X., Kurtan T., Wang B.-G. (2014). Varioxepine A, a 3 H-oxepine-containing alkaloid with a new oxa-cage from the marine algal-derived endophytic fungus *Paecilomyces variotii*. Org. Lett..

[27] Dasanayaka S., Nong X.-H., Liang X., Liang J.-Q., Amin M., Qi S.-H. (2019). New dibenzodioxocinone and pyran-3, 5-dione derivatives from the deep-sea-derived fungus *Penicillium canescens* SCSIO z053. J. Asian Nat. Prod. Res..

[28] Ren J., Xue C., Tian L., Xu M., Chen J., Deng Z., Proksch P., Lin W. (2009). Asperelines A− F, peptaibols from the marine-derived fungus *Trichoderma asperellum*. J. Nat. Prod..

[29] Jiang W., Ye P., Chen C.-T., Wang K., Liu P., He S., Wu X., Gan L., Ye Y., Wu B. (2013). Two novel hepatocellular carcinoma cycle inhibitory cyclodepsipeptides from a hydrothermal vent crab-associated fungus *Aspergillus clavatus* C2WU. Mar. Drugs.

[30] Luo X.W., Yun L., Liu Y.J., Zhou X.F., Liu Y.H. (2019). Peptides and polyketides isolated from the marine sponge-derived fungus *Aspergillus terreus* SCSIO 41008. Chin. J. Nat. Med..

[31] Liang X., Zhang X.-Y., Nong X.-H., Wang J., Huang Z.-H., Qi S.-H. (2016). Eight linear peptides from the deep-sea-derived fungus *Simplicillium obclavatum* EIODSF 020. Tetrahedron.

[32] Uchida R., Nakajyo K., Kobayashi K., Ohshiro T., Terahara T., Imada C., Tomoda H. (2016). 7-Chlorofolipastatin, an inhibitor of sterol O-acyltransferase, produced by marine-derived *Aspergillus ungui* NKH-007. J. Antibiot..

[33] Pan C., Shi Y., Auckloo B.N., ul Hassan S.S., Akhter N., Wang K., Ye Y., Chen C.-T.A., Tao X., Wu B. (2017). Isolation and antibiotic screening of fungi from a hydrothermal vent site and characterization of secondary metabolites from a *Penicillium* isolate. Mar. Biotechnol..

[34] Huang Z., Nong X., Ren Z., Wang J., Zhang X., Qi S. (2017). Anti-HSV-1, antioxidant and antifouling phenolic compounds from the deep-sea-derived fungus *Aspergillus versicolor* SCSIO 41502. Bioorg. Med. Chem. Lett..

[35] Liu C.-C., Zhang Z.-Z., Feng Y.-Y., Gu Q.-Q., Li D.-H., Zhu T.-J. (2019). Secondary metabolites from Antarctic marine-derived fungus *Penicillium crustosum* HDN153086. Nat. Prod. Res..

[36] Orfali R.S., Aly A.H., Ebrahim W., Abdel-Aziz M.S., Müller W.E., Lin W., Daletos G., Proksch P. (2015). Pretrichodermamide C and N-methylpretrichodermamide B, two new cytotoxic epidithiodiketopiperazines from hyper saline lake derived *Penicillium* sp.. Phytochem. Lett..

[37] Fan Z., Sun Z.-H., Liu Z., Chen Y.-C., Liu H.-X., Li H.-H., Zhang W.-M. (2016). Dichotocejpins A–C: New diketopiperazines from a deep-sea-derived fungus *Dichotomomyces cejpii* FS110. Mar. Drugs.

[38] Li Y., Ye D., Shao Z., Cui C., Che Y. (2012). A sterol and spiroditerpenoids from a *Penicillium* sp. isolated from a deep sea sediment sample. Mar. Drugs.

[39] Li X.-D., Li X.-M., Li X., Xu G.-M., Liu Y., Wang B.-G. (2016). Aspewentins D–H, 20-nor-isopimarane derivatives from the deep sea sediment-derived fungus *Aspergillus wentii* SD-310. J. Nat. Prod..

[40] Wu G., Lin A., Gu Q., Zhu T., Li D. (2013). Four new chloro-eremophilane sesquiterpenes from an Antarctic deep-sea derived fungus, *Penicillium* sp. PR19N-1. Mar. Drugs.

[41] Lin A., Wu G., Gu Q., Zhu T., Li D. (2014). New eremophilane-type sesquiterpenes from an antarctic deep-sea derived fungus, *Penicillium* sp. PR19 N-1. Arch. Pharm. Res..

[42] Zhou Y., Li Y.-H., Yu H.-B., Liu X.-Y., Lu X.-L., Jiao B.-H. (2018). Furanone derivative and sesquiterpene from antarctic marine-derived fungus *Penicillium* sp. S-1-18. J. Asian Nat. Prod. Res..

[43] Li X.-D., Li X., Li X.-M., Xu G.-M., Zhang P., Meng L.-H., Wang B.-G. (2016). Tetranorlabdane diterpenoids from the deep sea sediment-derived fungus *Aspergillus wentii* SD-310. Planta Med..

[44] Li X., Li X.-M., Li X.-D., Xu G.-M., Liu Y., Wang B.-G. (2016). 20-Nor-isopimarane cycloethers from the deep-sea sediment-derived fungus *Aspergillus wentii* SD-310. RSC Adv..

[45] Fang W., Lin X., Zhou X., Wan J., Lu X., Yang B., Ai W., Lin J., Zhang T., Tu Z. (2014). Cytotoxic and antiviral nitrobenzoyl sesquiterpenoids from the marine-derived fungus *Aspergillus ochraceus* Jcma1F17. MedChemComm.

[46] Niu S., Fan Z.-W., Xie C.-L., Liu Q., Luo Z.-H., Liu G., Yang X.-W. (2017). Spirograterpene A, a tetracyclic spiro-diterpene with a fused 5/5/5/5 ring system from the deep-sea-derived fungus *Penicillium granulatum* MCCC 3A00475. J. Nat. Prod..

[47] Chung Y.-M., Wei C.-K., Chuang D.-W., El-Shazly M., Hsieh C.-T., Asai T., Oshima Y., Hsieh T.-J., Hwang T.-L., Wu Y.-C. (2013). An epigenetic modifier enhances the production of anti-diabetic and anti-inflammatory sesquiterpenoids from *Aspergillus sydowii*. Bioorg. Med. Chem..

[48] Li C.-S., Li X.-M., Gao S.-S., Lu Y.-H., Wang B.-G. (2013). Cytotoxic anthranilic acid derivatives from deep sea sediment-derived fungus *Penicillium paneum* SD-44. Mar. Drugs.

[49] Chen L., Gong M.-W., Peng Z.-F., Zhou T., Ying M.-G., Zheng Q.-H., Liu Q.-Y., Zhang Q.-Q. (2014). The marine fungal metabolite, dicitrinone B, induces A375 cell apoptosis through the ROS-related caspase pathway. Mar. Drugs.

[50] Pan C., Shi Y., Chen X., Chen C.-T.A., Tao X., Wu B. (2017). New compounds from a hydrothermal vent crab-associated fungus *Aspergillus versicolor* XZ-4. Org. Biomol. Chem..

[51] Xu R., Xu G.-M., Li X.-M., Li C.-S., Wang B.-G. (2015). Characterization of a newly isolated marine fungus *Aspergillus dimorphicus* for optimized production of the anti-tumor agent wentilactones. Mar. Drugs.

